# Erythritol inhibits the growth of periodontal-disease-associated bacteria isolated from canine oral cavity

**DOI:** 10.1016/j.heliyon.2022.e10224

**Published:** 2022-08-13

**Authors:** Mamu Shimizu, Shingo Miyawaki, Taishin Kuroda, Miyu Umeta, Mifumi Kawabe, Kazuhiro Watanabe

**Affiliations:** Laboratory of Veterinary Surgery, Faculty of Applied Biological Sciences, Gifu University, 1-1 Yanagido, Gifu 501-1193, Japan

**Keywords:** Periodontal disease, Dogs, Erythritol, *Porphyromonas gulae*, *Porphyromonas macacae*

## Abstract

Periodontal disease (PD) is the most common oral disease that is caused by infection with periodontal-disease-associated bacteria (PDAB) such as *Porphyromonas gulae* and *Porphyromonas macacae* in dogs as well as in humans. Unlike humans, most dogs do not follow daily oral hygiene routine, and this results in many dogs being affected by PD. Thus, to prevent PD, it is important to control PDAB. Xylitol is a sugar alcohol that inhibits the growth of oral bacteria in humans. However, xylitol is poisonous to dogs and can lead to hypoglycemia and hepatic failure. Herein, we show the inhibitory effect of erythritol, a sugar alcohol that can be used safely in dogs, on the growth of PDAB isolated from dogs with PD. Oral bacteria were isolated from the oral cavities of dogs with PD, and the distribution of PDAB was evaluated. Interestingly, *Porphyromonas gingivalis*, a bacterium typically responsible for PD in humans, was not isolated from dog samples. The bacteriostatic effect of erythritol supplementation was investigated on isolated PDAB *in vitro*. Our results show that erythritol exert bacteriostatic effects on PDAB comparable to xylitol. Thus, application of erythritol can be suggested to control PDAB in dogs in the future.

## 1 Introduction

Periodontal disease (PD) is one of the most common oral diseases in both humans and dogs [1, 2]. PD is characterized by gingivitis, a reversible condition involving gingival inflammation, and periodontitis, an irreversible condition resulting in the destruction of periodontal tissues including the cementum, periodontal ligament, and alveolar bone. Periodontitis is a complex infection as various factors play causative roles simultaneously [1, 3, 4]. To prevent PD, it is important to practice good oral hygiene, including brushing teeth daily [5, 6, 7]. Almost all dogs are affected by PD at some point in their lifetime, as it is difficult to maintain a daily oral hygiene routine in dogs [8, 9]. As a consequence, dogs with severe PD require surgical treatment, which is invasive and requires anesthesia [1, 10]. Therefore, it is critical to develop effective methods to prevent PD in dogs.

Periodontal-disease-associated bacteria (PDAB) are present in plaque and tartar on the teeth. These bacteria cause gingivitis, which is followed by the spread of inflammation to the tissues supporting the teeth, resulting in periodontitis as PD progresses [1, 4]. Therefore, dental plaque is an important target in the prevention of PD. To reduce plaque, it is necessary to inhibit the bacteria that form it (11). Pathogenic oral bacteria such as *Porphyromonas gingivalis* and *Porphyromonas gulae* are the majorPDAB [11, 12]. Recently, *Porphyromonas macacae* (also called *Porphyromonas salivosa*) was also isolated from the oral cavity of dogs with PD [13, 14, 15]. It has been proposed that controlling these *Porphyromonas* spp. may inhibit plaque formation and suppress the progression of PD [15, 16]. Therefore, inhibiting the growth of these bacteria could be an effective strategy to prevent PD.

Several studies have conclusively established that sugar alcohols, such as xylitol, inhibit the growth of pathogenic oral bacteria, including cariogenic microorganisms in humans [17, 18, 19]. Sugar alcohols have bacteriostatic effects on a variety of bacteria [20, 21], and they are effectively and widely used in daily oral care in humans [22, 23]. However, in dogs, xylitol cannot be used because it results in life-threatening hypoglycemia and acute liver failure [24, 25, 26, 27, 28]. Erythritol, a sugar alcohol similar to xylitol, is not metabolized and undergoes efficient renal excretion; therefore, it is unlikely to result in hypoglycemia [29, 30, 31]. Thus, erythritol has been reported to be safe for use in dogs [31]. Erythritol may be a candidate agent to prevent PD in dogs; however, there is no evidence that erythritol inhibits the growth of PDAB [32].

Thus, the objective of this study was to investigate whether erythritol exerts bacteriostatic effects on PDAB isolated from dogs with PD and can be used to treat periodontitis in dogs.

## 2 Methods

### 2.1 Isolation and cultivation of bacteria from oral swab specimens

Using sterile swabs, dental plaque specimens were collected from the gingival margin of the right and left fourth maxillary premolar of four dogs with PD. The number of bacteria in dental plaque has been reported to increase with the use of the maxillary fourth premolar, and this tooth was also used in this experiment [7]. The experiment was performed on dogs that had been diagnosed by a veterinarian with stage 4 PD according to American Veterinary Dental College criteria of PD [33]. The experimental procedures were performed according to the guidelines for the care and use of laboratory animals approved by the Animal Care and Use Committee of Gifu University (permission numbers: 2020-16, 2021-99).

The samples were cultured anaerobically for 48 h at 37 °C in modified Gifu anaerobic medium (GAM) broth (NISSUI, Tokyo, Japan). AneroPack square jars and AneroPack - Kenki (Mitsubishi Gas Chemical Company, Inc., Tokyo, Japan) were used for the anaerobic culture. These pre-isolated cultures represented a mixture of different oral bacteria from the dog oral cavity and are referred to as mixed cultures in this study. Subsequently, to isolate black-pigmented anaerobic bacteria as the primary periopathogen [34, 35], the mixed culture was incubated anaerobically for 48 h at 37 °C on anaerobic blood hemin vitamin K (ABHK) blood agar medium (NISSUI, Tokyo, Japan). Black-pigmented colonies growing on the ABHK agar medium, suspected to belong to the *Porphyromonas* genus, were collected and cultured anaerobically in modified GAM broth (Fig. S1A) [34, 35].

### 2.2 Identification of the bacterial strains

Bacterial DNA was extracted from the isolated black-pigmented colonies using an alkaline treatment and heating method. Briefly, 50 μL of 50 mM NaOH was added to the bacterial pellet, and the mixture was incubated at 95 °C for 30 min. Subsequently, 12.5 μL of 1 M Tris-HCl (pH 7.5) was added, and the solution was mixed well. PCR was performed on the extracted DNA to identify the periodontopathogens. We designed 16S rRNA gene-based primers for *P. gulae* or *P. macacae* using the nucleotide sequence of the 16S rRNA gene from *P. gulae* ATCC 51700 and *P. macacae* ATCC 33141. The targeted DNA sequences distinguishable between *P. gulae* and *P. macacae* are shown in Fig. S1B. PCR amplification was performed using KOD One (Toyobo, Osaka, Japan) under the following conditions: an initial denaturation at 95 °C for 30 s, followed by 30 cycles at 98 °C for 10 s, 60 °C for 5 s, and 68 °C for 15 s. The PCR products were resolved via electrophoresis using a 2.5% agarose gel, and the amplicon size was determined (Fig. S1C). If no amplicon of appropriate size was observed with either primer set, PCR was repeated with universal primers (27F/519R) for bacterial 16S rRNA, and amplicons were subjected to agarose gel electrophoresis [36]. PCR products were extracted from the agarose gel using the FastGene gel/PCR extraction kit (NIPPON Genetics, Tokyo, Japan), according to the manufacturer's instructions. The extracted PCR products were sequenced, and the results were analyzed using a BLAST search to identify bacteria with matching sequences. The primers used are listed in Table S1.

### 2.3 Bacterial growth study

Xylitol (Sigma, St. Louis, MO, USA) or erythritol (Sigma, St. Louis, MO, USA) was added to the modified GAM broth, which was sterilized by filtration (Sartorius, Gottingen, Germany). The medium contained 0.01%, 0.02%, 0.1%, 1%, 5%, or 10% (w/v) xylitol or erythritol, while the corresponding control medium excluded both. Clindamycin, a lincomycin antibiotic, was used as an experimental positive control at concentrations of 1.5 × 10, 1.5 × 10^2^, or 1.5 × 10^3^ μg/mL. Each strain was cultured anaerobically in an appropriate basic medium at 37 °C. To verify the bacteriostatic effect of erythritol, bacterial proliferation was evaluated in modified GAM broth supplemented with sugar alcohols. Four mixed cultures and eight isolated samples (four samples each of *P. gulae* and *P. macacae*) were used in this study. The isolated samples were identified via sequence analysis. Each strain was inoculated (100 CFU/mL) in modified GAM broth supplemented with sugar alcohol at various concentrations or control medium. The culture was carried out in 96-well flat-bottomed microplates, and bacterial proliferation was evaluated indirectly by measuring the optical density (OD) of the medium. The anaerobic culture was incubated for 24 h at 37 °C, and the OD of each well was measured every 4 h at 620 nm using Multiskan FC (Thermo Fisher Science, Waltham, MA, USA). The OD of the negative control was subtracted from each value to correct for the background effect of the broth.

### 2.4 Effect of glucose on the bacteriostatic effect of erythritol

Appropriate concentrations of erythritol (Sigma, St. Louis, MO, USA) and glucose (Kanto Chemical, Nihonbashi, Tokyo, Japan) were added to heart infusion broth containing hemin (10 mg/L) and menadione (5 mg/L) and sterilized by filtration (Saltorius, Gottingen, Germany). In addition to 1% erythritol, the medium was prepared with 0.005%, 0.01%, 0.025%, 0.05%, or 0.1% glucose and without glucose (control). Four mixed cultures and eight isolated samples (four samples each of *P. gulae* and *P. macacae*) were used in this study. Each strain was inoculated (100 CFU/mL) in the medium containing hemin, menadione, and erythritol at constant concentrations and glucose at variable concentrations. The culture was carried out in 96-well flat-bottomed microplates, and the proliferation of bacteria was evaluated indirectly by measuring the OD of the medium. The anaerobic culture was incubated for 24 h at 37 °C, and the OD of each well was measured every 4 h in the same way as for the bacterial growth experiment.

### 2.5 Statistical analysis

Power analysis and sample size estimation were performed using the G∗ power software (version 3.1.9.2) (Effect size: 0.25, α error: 0.05, power: 0.9). Statistical analyses were performed using the R software (version 3.6.3). Tukey's test (*p* < 0.05) was performed when a significant difference was found using one-way ANOVA test (*p* < 0.05).

## 3 Results

### 3.1 Isolation of *P. gulae* and *P. macacae* from dogs with PD

We cultured oral swab specimens collected from the gingival margin of the right fourth maxillary premolar. Black-pigmented anaerobic bacteria were isolated from blood agar cultures (Fig. S1). To identify the bacterial strains, PCR was performed using genomic DNA extracted from the isolated clones. Among the 103 isolated black-pigmented colonies, 48 (50.5%) were determined to be *P. gulae,* and 39 (41.1%) were determined to be *P. macacae* (Table 1). More than 90% of black-pigmented anaerobic bacteria were either *P. gulae* or *P. macacae,* which are common PDAB in dogs. The other isolated bacteria were unidentified bacteria or an unidentified *Bacteroides* genus. *P. gingivalis*, which has been reported as a PDAB in humans, was not detected in the dogs used in this study. Thus, we successfully isolated PDAB from dogs with PD.

**Table 1:** Contents of black-pigmented colonies isolated from the oral cavity of dogs.

	*Porphyromonas gulae*	*Porphyromonas macacae*	*Bacteroides denticanum*	*Bacteroides heparinolyticus*	Unknown
Beagle 1	11	4	1	0	4
Beagle 2	8	14	1	0	0
Chihuahua	14	8	0	1	1
Toy Poodle	15	13	0	0	0
Total	48	39	2	1	5
%	50.5	41.1	2.1	1.0	5.3

### 3.2 PDAB growth inhibition at different erythritol concentrations

**Figure 1:**
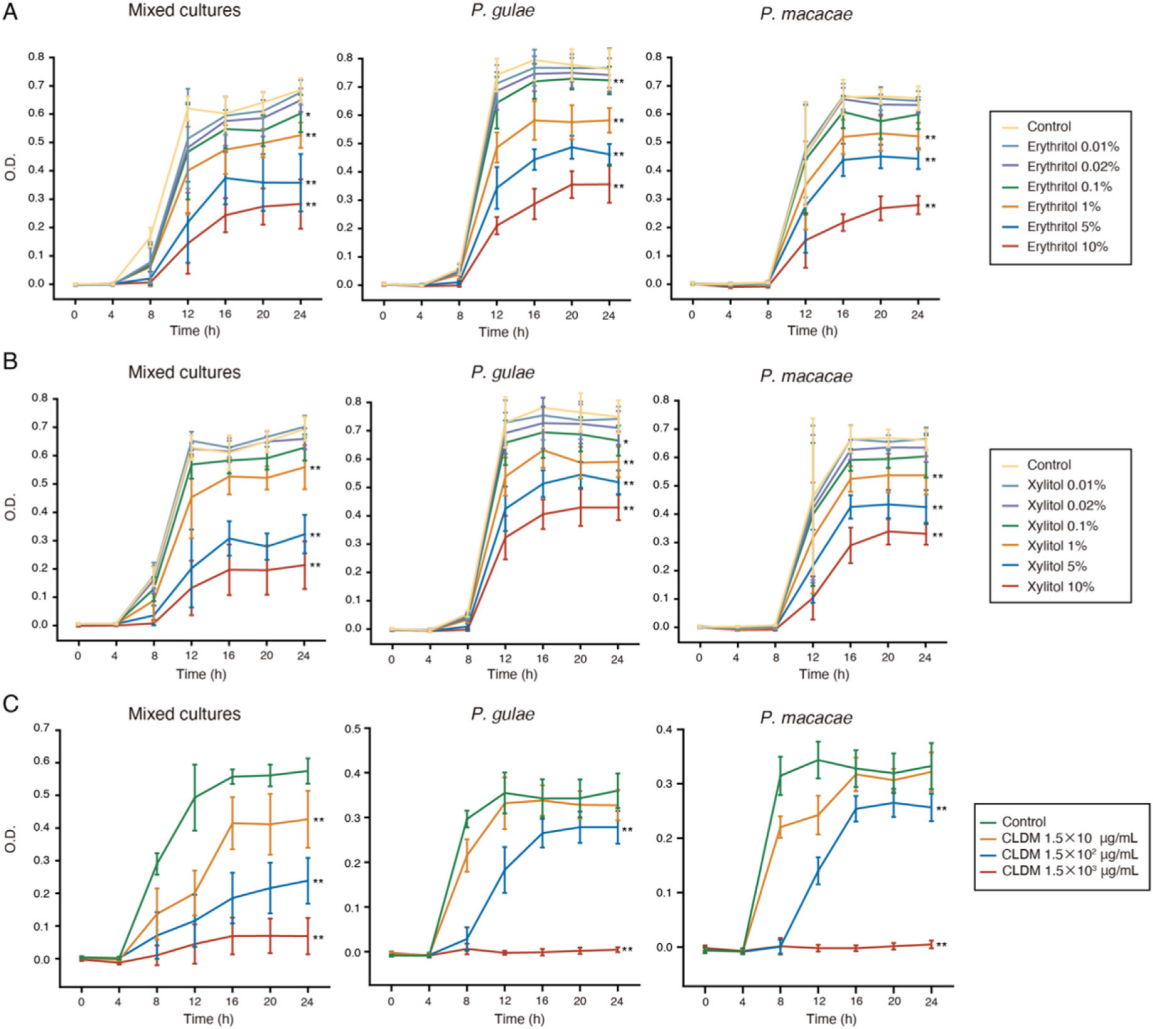
Bacteriostatic effect of erythritol, xylitol, and clindamycin on the pathogenic oral bacteria isolated from dogs. Growth of oral bacteria in media supplemented with erythritol (A), xylitol (B), and clindamycin (C). The optical density (OD) of each tube was measured throughout the bacterial culture, at 620 nm against the standard medium. The OD values were calculated as the means ± S.D. of at least three measurements (n = 4 clones). CLDM represents clindamycin. (∗*p* < 0.05, ∗∗*p* < 0.01 between the indicated concentration and control at 24 h, one-way analysis of variance [ANOVA] with Tukey's post hoc test).

To evaluate the bacteriostatic effect of erythritol on oral bacteria, the growth of the isolated PDAB was investigated at various erythritol concentrations. Xylitol was used at the same concentrations for comparison. The growth of pre-isolated mixed cultures was inhibited by erythritol and xylitol, indicating that erythritol is effective in inhibiting the growth of different oral bacteria (Figure 1). In pre-isolated mixed cultures, there were significant differences at erythritol concentrations of 0.1% (*p* < 0.05) and over 1% (*p* < 0.01) compared with the control and there were also significant differences at xylitol concentrations over 1% (*p* < 0.01) compared with the control. After 24 h of incubation, 5% and 10% erythritol inhibited *P. gulae* growth by 30% and 41%, respectively. Similarly, after 24 h of incubation, 5% and 10% xylitol resulted in 23% and 32% growth inhibition, respectively (Figure 1). In *P. gulae*, there were significant differences at erythritol concentrations over 0.1% (*p* < 0.01) compared with the control, and there were also significant differences at xylitol concentrations of 0.1% (*p* < 0.05) and over 1% (*p* < 0.01) compared with the control. After 24 h of incubation, 5% and 10% erythritol inhibited *P. macacae* growth by 23% and 38%, respectively. Similarly, 5% and 10% xylitol resulted in 23% and 33% growth inhibition, respectively, after 24 h of incubation (Figure 1). The minimum concentration of erythritol at which growth inhibition was observed in both *P. gulae* and *P. macacae* was 1%. In *P. macacae*, there were significant differences at erythritol and xylitol concentrations over 1% (*p* < 0.01) compared with the control. Neither xylitol nor erythritol could inhibit the growth of the bacteria as completely as the antibiotic clindamycin. Notably, following the 24 h incubation with erythritol, the bacterial concentration decreased in a concentration-dependent manner. These results indicate that erythritol, as well as xylitol, inhibit the growth of *P. gulae* and *P. macacae* isolated from the oral cavity of dogs with PD.

### 3.3 Glucose supplementation inhibits bacteriostatic effects of erythritol on PDAB growth

**Figure 2:**
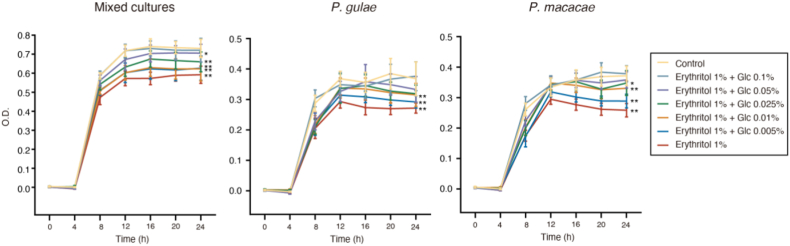
Bacteriostatic effects of erythritol on periodontal-disease-associated bacteria in the presence of glucose. Effects of erythritol on mixed cultures, and *Porphyromonas gulae* and *Porphyromonas macacae* growth in heart infusion medium supplemented with 1% erythritol and various glucose concentrations. The OD values were calculated as the means ± S.D. of at least three measurements (n = 4 clones). (∗*p* < 0.05, ∗∗*p* < 0.01 between the indicated concentration and control at 24 h, one-way analysis of variance [ANOVA] with Tukey's post hoc test).

To evaluate the inhibitory effect of erythritol on PDAB in the presence of glucose, bacterial growth was evaluated following incubation with 1% erythritol supplemented with glucose at various concentrations. Because the glucose-rich modified GAM medium was not suitable for this experiment, heart infusion medium with menadion and hemin was used. The bacteriostatic effect of erythritol was inhibited in the presence of glucose in all groups (Figure 2), and the bacteriostatic effect of erythritol was abrogated in the presence of 0.05% glucose. These data indicate that glucose interferes with the bacteriostatic effect of erythritol on PDAB.

## 4 Discussion

Erythritol may be used to prevent PD if its effectiveness against PDAB is clarified. To this end, we cultured *P. gulae* and *P. macacae* anaerobically with different dilutions of erythritol in liquid medium to confirm its effect on PDAB in dogs. In this study, *P. gingivalis*, which is typically detected in humans with periodontitis, was not detected (Table 1). This is consistent with previous report that *P. gulae* and *P. macacae* are more prevalent than *P. gingivalis* in the periodontal tissues of dogs affected by PD [14, 34]. This indicates that although erythritol inhibits the growth of *P. gingivalis* [37], the inhibition of *P. gulae* and *P. macacae* may be more effective to prevent PD in dogs.

Our results showed that erythritol markedly inhibited the growth of pathogenic oral bacteria isolated from dogs with PD in a concentration-dependent manner. The degree of growth inhibition exhibited by xylitol and erythritol was comparable, indicating that like xylitol, erythritol inhibits the growth of both *P. gulae* and *P. macacae*. *P. gulae*, detected in the oral cavity of humans with gingivitis or chronic periodontitis, and several other species may be transmitted during close daily contact with dogs [38, 39]. Erythritol is effective in inhibiting the growth of *P. gulae* and *P. macacae*, PDAB of dogs isolated in this study.

The effect of glucose on the inhibitory effect of erythritol on bacterial growth was determined with 1% erythritol in the medium (minimum concentration at which inhibition was observed in the two isolated bacteria). The inhibitory effect of erythritol was counteracted by the addition of glucose. Glucose is reported to repress the transport and catabolic activities of sugar alcohols such as xylitol [18]. This explains the outcome of our study, and thus, the mode of erythritol-mediated inhibition of *P. gulae* and *P. macacae* is similar and resembles that of xylitol [18]. Our results provide evidence that the bacteriostatic effect of erythritol may be diminished in an oral environment with high glucose levels.

Our results show that the growth of *P. gulae* and *P. macacae*, which are the typical PDAB found in dogs, was inhibited by erythritol in a concentration-dependent manner, suggesting that the use of erythritol is useful for reducing these bacteria. To use erythritol in dogs for treatment of periodontitis in the future, it is necessary to establish a method of providing erythritol to dogs to prevent PD and demonstrate its effects *in vivo*. There are several possible ways to administer erythritol to dogs. One is to apply a toothpaste or gel containing erythritol to plaque-covered tooth surface. This method would ensure complete contact between the plaque and erythritol. It may also be effective to include erythritol in the composition of dental gum. Another method is to dilute erythritol in water and give it to the dog. This is an easy method to implement, but it is necessary to verify that erythritol is effective in contacting the plaque. In this case, even if erythritol is administered at 1–10% concentration, the range in which bacterial growth is inhibited, there is no toxicity to dogs [31]. There are some limitations to this study. In this study, the growth inhibitory effect of erythritol was confirmed for two bacterial species, *P. gulae* and *P. macacae*, isolated from dogs with PD, but not for other PDAB. In addition, the model was a simple one in which erythritol was diluted in the culture medium. Further investigation of the effect of erythritol in a complex model using dental plaque or in actual dogs is needed. Nevertheless, the results of the current study clearly show that erythritol inhibits the growth of *P. gulae*, the main causative agent of PD in dogs. In the future, erythritol could be used effectively in daily oral care for the management of PD in dogs.

## Declarations

### Author contribution statement

Shingo Miyawaki: Conceived and designed the experiments; Performed the experiments; Analyzed and interpreted the data; Wrote the paper.

Kazuhiro Watanabe: Conceived and designed the experiments.

Mamu Shimizu, Mifumi Kawabe: Performed the experiments; Analyzed and interpreted the data; Wrote the paper.

Taishin Kuroda, Miyu Umeta: Performed the experiments; Analyzed and interpreted the data.

### Funding statement

This work was supported by KAKENHI from the Japan Society for the Promotion of Science (https://www.jsps.go.jp/) Grant Numbers 21H02358 (SM). SM was supported by the ​Takeda Science Foundation and ​Mitsubishi Foundation.

### Data availability statement

No data was used for the research described in the article.

### Declaration of interests statement

The authors declare no conflict of interest.

### Additional information

No additional information is available for this paper.

## Appendix A Supplementary data

Supplementary files.docx Supplementary files.docx

## References

[1] Niemiec B.A. (2008). Periodontal disease. Top. Companion Anim. Med..

[2] Albandar J.M., Brunelle J.A., Kingman A. (1999). Destructive periodontal disease in adults 30 Years of age and older in the United States, 1988-1994. J. Periodontol..

[3] Cekici A., Kantarci A., Hasturk H., van Dyke T.E. (2014). Inflammatory and immune pathways in the pathogenesis of periodontal disease. Periodontology 2000.

[4] Kinane D.F. (2001). Causation and pathogenesis of periodontal disease. Periodontology 2000.

[5] Ingham K.E., Gorrel C. (2001). Effect of long-term intermittent periodontal care on canine periodontal disease. J. Small Anim. Pract..

[6] Tromp J.A.H., Jansen J., Pilot T. (1986). Gingival health and frequency of tooth brushing in the beagle dog model. Clinical findings. J. Clin. Periodontol..

[7] Watanabe K., Hayashi K., Kijima S., Nonaka C., Yamazoe K. (2015). Tooth brushing inhibits oral bacteria in dogs. J. Vet. Med. Sci..

[8] Wallis C., Marshall M., Colyer A., O’Flynn C., Deusch O., Harris S. (2015). A longitudinal assessment of changes in bacterial community composition associated with the development of periodontal disease in dogs. Vet. Microbiol..

[9] Glickman L.T., Glickman N.W., Moore G.E., Lund E.M., Lantz G.C., Pressler B.M. (2011). Association between chronic azotemic kidney disease and the severity of periodontal disease in dogs. Prev. Vet. Med..

[10] Niemiec B.A. (2008). Extraction techniques. Top. Companion Anim. Med..

[11] Lenzo J.C., O’Brien-Simpson N.M., Orth R.K., Mitchell H.L., Dashper S.G., Reynolds E.C. (2016). *Porphyromonas gulae* has virulence and immunological characteristics similar to those of the human periodontal pathogen *Porphyromonas gingivalis*. Infect. Immun..

[12] Kato Y., Shirai M., Murakami M., Mizusawa T., Hagimoto A., Wada K., Nomura R., Nakano K., Ooshima T., Asai F. (2011). Molecular detection of human periodontal pathogens in oral swab specimens from dogs in Japan. J. Vet. Dent..

[13] Fournier D., Mouton C., Lapierre P., Kato T., Okuda K., Ménard C. (2001). Porphyromonas gulae sp. nov., an anaerobic, gram-negative coccobacillus from the gingival sulcus of various animal hosts. Int. J. Syst. Evol. Microbiol..

[14] do Nascimento Silva A., de Avila E.D., Nakano V., Avila-Campos M.J. (2017). Pathogenicity and genetic profile of oral Porphyromonas species from canine periodontitis. Arch. Oral Biol..

[15] Koyota Y., Watanabe K., Toyama T., Sasaki H., Hamada N. (2019). Purification and characterization of a fimbrial protein from Porphyromonas salivosa ATCC 49407. J. Vet. Med. Sci..

[16] Inaba K., Koyota Y., Sasaki H., Toyama T., Hiramine H., Ogawa T., Watanabe K., Hamada N. (2019). Porphyromonas salivosa ATCC 49407 fimbriae induced osteoclast differentiation and cytokine production. J. Kanagawa Odontol. Soc..

[17] Na H.S., Kim S.M., Kim S., Choi Y.H., Chung J. (2013). Effect of xylitol on various oral bacteria. Int. J. Oral Biol.

[18] Chan A., Ellepola K., Truong T., Balan P., Koo H., Seneviratne C.J. (2020). Inhibitory effects of xylitol and sorbitol on Streptococcus mutans and Candida albicans biofilms are repressed by the presence of sucrose. Arch. Oral Biol..

[19] Badet C., Furiga A., Thébaud N. (2008). Effect of xylitol on an in vitro model of oral biofilm. Oral Health Prev. Dent..

[20] Piao J., Kawahara-Aoyama Y., Inoue T., Adachi S. (2016). Bacteriostatic activities of monoacyl sugar alcohols against thermophilic sporeformers. Biosci., Biotechnol., Biochem..

[21] Ali S.G. (2017). In Vitro synergistic effect of xylitol with Salvadora presica L. extracts and cephalexin on Streptococcus mutans strains. Int. J. Adv. Biomed.

[22] Alanen P., Isokangas P., Gutmann K. (2000). Xylitol candies in caries prevention: results of a field study in Estonian children. Community Dent. Oral Epidemiol..

[23] Haresaku S., Hanioka T., Tsutsui A., Yamamoto M., Chou T., Gunjishima Y. (2007). Long-term effect of xylitol gum use on mutans streptococci in adults. Caries Res..

[24] Xia Z., He Y., Yu J. (2009). Experimental acute toxicity of xylitol in dogs. J. Vet. Pharmacol. Therapeut..

[25] Dunayer E.K., Gwaltney-Brant S.M. (2006). Acute hepatic failure and coagulopathy associated with xylitol ingestion in eight dogs. J. Am. Vet. Med. Assoc..

[26] Schmid R.D., Hovda L.R. (2016). Acute hepatic failure in a dog after xylitol ingestion. J. Med. Toxicol..

[27] Todd J.M., Powell L.L. (2007). Xylitol intoxication associated with fulminant hepatic failure in a dog. J. Vet. Emerg. Crit. Care.

[28] Hirata Y., Fujisawa M., Sato H., Asano T., Katsuki S. (1966). Blood glucose and plasma insulin responses to xylitol administrated intravenously in dogs. Biochem. Biophys. Res. Commun..

[29] Munro I.C., Bernt W.O., Borzelleca J.F., Flamm G., Lynch B.S., Kennepohl E., Bär E.A., Modderman J. (1998). Erythritol: an interpretive summary of biochemical, metabolic, toxicological and clinical data. Food Chem. Toxicol..

[30] Yokozawa T., Kim H.Y., Cho E.J. (2002). Erythritol attenuates the diabetic oxidative stress through modulating glucose metabolism and lipid peroxidation in streptozotocin-induced diabetic rats. J. Agric. Food Chem..

[31] Eapen A.K., de Cock P., Crincoli C.M., Means C., Wismer T., Pappas C. (2017). Acute and sub-chronic oral toxicity studies of erythritol in Beagle dogs. Food Chem. Toxicol..

[32] de Cock P., Mäkinen K., Honkala E., Saag M., Kennepohl E., Eapen A. (2016). Erythritol is more effective than xylitol and sorbitol in managing oral health endpoints. International J. Dent..

[33] Wolf H.F., Rateitschak E.M., Rateitschak K.H., Hassell T.M. (2005).

[34] Hardham J., Dreier K., Wong J., Sfintescu C., Evans R.T. (2005). Pigmented-anaerobic bacteria associated with canine periodontitis. Vet. Microbiol..

[35] Yamasaki T., Nagata A., Kiyoshige T., Sato M., Nakamura R. (1990). Black-pigmented, asaccharolytic Bacteroides species resembling Porphyromonas gingivalis (Bacteroides gingivalis) from beagle dogs. Oral Microbiol. Immunol..

[36] Lane D.J., Stackebrandt E., Goodfellow M. (1991). Nucleic Acid Techniques in Bacterial Systematics.

[37] Hashino E., Kuboniwa M., Alghamdi S.A., Yamaguchi M., Yamamoto R., Cho H., Amano A. (2013). Erythritol alters microstructure and metabolomic profiles of biofilm composed of Streptococcus gordonii and Porphyromonas gingivalis. Mol. Oral Microbiol..

[38] Gaetti-Jardim E., Pereira M.F., Vieira E.M.M., Schweitzer C.M., Okamoto A.C., Ávila-Campos M.J. (2015). Occurrence of periodontal pathogens in ethnic groups from a native Brazilian reservation. Arch. Oral Biol..

[39] Yamasaki Y., Nomura R., Nakano K., Naka S., Matsumoto-Nakano M., Asai F., Ooshima T. (2012). Distribution of periodontopathic bacterial species in dogs and their owners. Arch. Oral Biol..

