# Supplementary material for: Erythritol inhibits the growth of periodontal-disease-associated bacteria isolated from canine oral cavity
Source: Heliyon. 2022 Aug 13;8(8):e10224. doi: 10.1016/j.heliyon.2022.e10224 (PMC9424944; doi:10.1016/j.heliyon.2022.e10224)
Supplement: Supplementary files.docx [file mmc1.docx]

**Supplementary Materials**

**
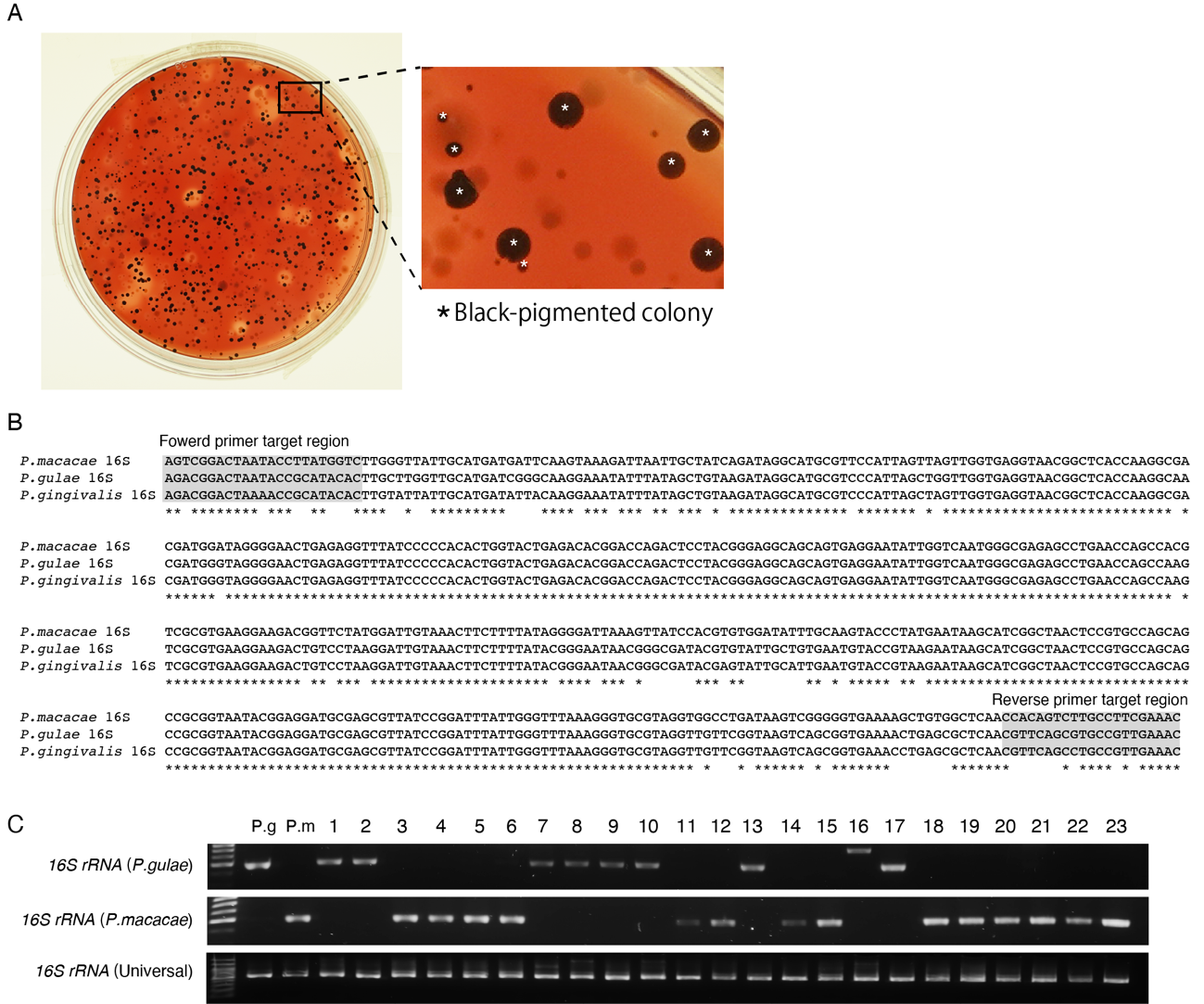
**

**Figure S1. Isolation and identification of bacterial strains from oral swab specimens.** Black-pigmented colonies in blood agar medium were picked, and bacterial species were identified by PCR and electrophoresis (A). PCR-amplified sequences and primer target regions (B). Asterisks represent nucleotides conserved between *Porphyromonas macacae,* *Porphyromonas gulae,* and *Porphyromonas gingivalis*. Gray background denotes primer target regions. Representative (Beagle 2) agarose gel electrophoresis images are shown (C). P.g: *P. gulae*; P.m: *P. macacae*.

**Table S1. Primers used in this study.**

| Target species | Primer sequence | Size (base pairs) | Reference |  |
| --- | --- | --- | --- | --- |
|  |  |  |  |  |
| Universal primer (positive control) | 5'-AGAGTTTGATCMTGGCTCAG-3' | 492 | 30 |  |
|  | 5'-GWATTACCGCGGCKGCTG-3' |  |  |  |
|  |  |  |  |  |
| *Porphyromonas gulae* | 5'-AGACGGACTAATACCGCATACAC-3' | 479 | This study |  |
|  | 5'-GTTTCAACGGCACGCTGAACG-3' |  |  |  |
|  |  |  |  |  |
| *Porphyromonas macacae* | 5'-AGTCGGACTAATACCTTATGGTC-3' | 479 | This study |  |
|  | 5'-GTTTCGAAGGCAAGACTGTGG-3' |  |  |  |

M=adenine or cytosine, W=adenine or thymine, K=guanine or thymine
